# Mechanistic Evaluation of LolA and *β*‐Sliding Clamp Inhibition by Silybin and Diclofenac as Gentamicin Adjuvants Against Enteropathogenic *Escherichia coli*

**DOI:** 10.1155/ijm/5624539

**Published:** 2026-09-28

**Authors:** Rafat Hossain Rafi, Anamul Haque, Md. Jonaid Tawfiq, Chandan Barai, Md. Rafiqul Islam Khan, Md. Omar Faruqe, Mst Sadia Afroz Shoily, Md. Abdul Alim Al-Bari

**Affiliations:** ^1^ Department of Pharmacy, Faculty of Science, University of Rajshahi, Rajshahi, Bangladesh, ru.ac.bd; ^2^ Department of Biochemistry and Molecular Biology, Faculty of Science, University of Rajshahi, Rajshahi, Bangladesh, ru.ac.bd; ^3^ Department of Computer Science and Engineering, Faculty of Engineering, University of Rajshahi, Rajshahi, Bangladesh, ru.ac.bd

**Keywords:** antibiotic adjuvant, diclofenac, enteropathogenic *E. coli*, gentamicin, molecular docking, molecular dynamics simulation, silybin

## Abstract

To address the challenges of antimicrobial resistance, one potential approach is to repurpose existing drugs. In the current study, structurally distinct antibiotics were combined with the repurposed drugs silybin and diclofenac to evaluate the potential of these combinations against resistant bacterial strains. The investigation encompassed in vitro determination of the minimum inhibitory concentration (MIC) and time‐kill kinetics, as well as in vivo assessments of percentage change in body weight, total white blood cell (WBC) count, colitis and edema, histopathological changes, stool scores, and blood culture in an enteropathogenic *Escherichia coli* (EPEC)‐infected mouse model. Both silybin and diclofenac potentiated the activity of several antibiotics, with gentamicin showing the most promising results. The gentamicin–silybin (Gen + Sily) and gentamicin–diclofenac (Gen + Dic) combinations not only demonstrated sustained antibacterial activity in the time‐kill assay compared with gentamicin monotherapy but also provided greater protection against crypt cell damage and more effectively alleviated colitis and edema in the EPEC‐infected mouse model. In silico molecular docking and 250‐ns molecular dynamics simulations hypothesized two potential targets implicated in antibiotic adjuvant activity: LolA and the *β*‐sliding clamp protein. Molecular docking and MM/PBSA analyses indicated that silybin and diclofenac exhibited stronger binding affinities and more favorable total binding free energies toward LolA and the *β*‐sliding clamp protein, respectively, than the corresponding reference compounds.

## 1. Introduction

Antimicrobial resistance (AMR) poses significant challenges to global healthcare. A total of 4.95 million deaths occurred because of AMR, where bacterial AMR was accountable for 1.27 million deaths [1]. Studies have indicated a rise in the global prevalence of multidrug‐resistant (MDR) bacteria, with MDR gram‐negative bacteria posing the most significant threat [2]. The World Health Organization (WHO) listed priority pathogens into three categories (critical, high, and medium) for the R&D of new antibiotics. For example*, Acinetobacter baumannii, Klebsiella pneumoniae, and Escherichia coli* were designated as critical priorities, whereas *Pseudomonas aeruginosa* and *Shigella* spp. were defined as high‐priority pathogens belonging to the gram‐negative bacteria group [3]. In contrast, the global report on antibacterial agents in preclinical and clinical development, published by the WHO in January 2024, stated that the antibiotic development pipeline had become stagnant, with a significant lack of novel antimicrobial classes to combat critical priority pathogens [4]. The majority of compounds in the pipeline were derivatives of existing antibiotics [5]. The situation became exacerbated as numerous pharmaceutical companies discontinued their antimicrobial research and development initiatives because of regulatory hurdles, economic challenges, and limited profit margins [6, 7]. If this situation persists, the human race will enter a postantibiotic era, resulting in elevated morbidity, mortality, and healthcare expenses. Surgeries, organ transplantation, and diagnostic tests would become increasingly precarious, necessitating expensive medications and prolonged hospital stays [8, 9].

Drug repurposing, referred to as drug repositioning or drug redirecting, offers an alternative strategy for combating antimicrobial‐resistant pathogens. Drug repurposing involves identifying novel therapeutic applications for existing, marketed drugs that have already been approved, thereby extending their benefits beyond their original intended use [10]. Nonantibiotic drugs initially developed for other therapeutic indications may possess intrinsic antimicrobial properties or enhance the efficacy of conventional antibiotics. Consequently, these agents can function as antibiotic adjuvants by augmenting antibiotic activity when administered in combination [11]. Such nonantibiotic agents enhance the antibiotic spectrum by disrupting intrinsic resistance mechanisms, including inhibition of antibiotic‐hydrolyzing enzymes, suppression of efflux pump activity, increased membrane permeability, prevention of biofilm formation, and modulation of multiple cellular pathways [12]. Drug repurposing efforts bypass a substantial portion of the discovery and preclinical phases, leading to accelerated development timelines, reduced development risks, and lower overall costs compared with traditional drug development. Furthermore, the availability of established pharmacodynamic, pharmacokinetic, and toxicity data enables these agents to be rapidly advanced into clinical trials [10, 13].

Developing novel antimicrobials against MDR gram‐negative bacteria is highly challenging because of their robust structure, composed of the outer membrane (OM), periplasmic peptidoglycan layer, inner membrane (IM), and efflux pumps, forming an impermeable barrier to many antibiotics. Lipoproteins play a critical role in the structural integrity of the OM. Integration of the newly synthesized lipoproteins into the OM is mediated through the Lol (lipoprotein OM localization) transport system. The periplasmic chaperone LolA is one of the core components of the lipoprotein trafficking system, which is essential for OM lipoprotein localization and, consequently, OM integrity in gram‐negative bacteria. Disruption of this system leads to enhanced antibiotic permeability [14, 15]. Another therapeutic target is the sliding clamp, a *β* subunit of DNA polymerase III, which encircles bacterial double‐stranded DNA and allows the core enzyme to replicate freely along DNA. Due to its crucial role in DNA replication and repair, the *β*‐sliding clamp is considered a prime target for antimicrobial drugs [16, 17]. Because both LolA and the *β*‐sliding clamp are essential, broadly conserved among gram‐negative pathogens, and structurally distinct from conventional antibiotic targets, LolA and the *β*‐sliding clamp are considered crucial antimicrobial target proteins [14, 16, 17].

The aforementioned findings inspired the investigation of the antimicrobial and synergistic activities of nonantibiotic compounds in combination with antibiotics against resistant pathogens. Initially, several repurposed drugs were screened in the laboratory, including aspirin (a nonsteroidal anti‐inflammatory drug‐NSAID), empagliflozin (an antidiabetic drug), diclofenac (a NSAID), amitriptyline (an antidepressant), ursodeoxycholic acid (a secondary bile acid), and silybin (a hepatoprotective agent). Based on the results of the zone of inhibition, the current study selected silybin and diclofenac for further evaluation through in vitro antimicrobial tests, an in vivo study using the enteropathogenic *Escherichia coli* (EPEC) mouse model, and an in silico molecular docking and molecular dynamics (MD) simulation study.

## 2. Materials and Methods

### 2.1. Chemicals

The chemicals were of analytical reagent quality and were acquired from Sigma Chemical Co. (St. Louis, Missouri, United States). Drugs were obtained from different pharmaceutical companies: gentamicin, chloramphenicol, azithromycin, oxytetracycline, ciprofloxacin, diclofenac (Incepta Pharmaceuticals Ltd., Bangladesh); doxycycline, penicillin, erythromycin, and silybin (Square Pharmaceuticals Ltd., Bangladesh).

### 2.2. Bacterial Strains

Three different bacterial isolates were obtained from the Microbiology department′s culture collection at the icddr,b in Dhaka, Bangladesh: *P. aeruginosa* (ATCC 27853), *E. coli* (enteropathogenic strain), and *Shigella boydii* (ATCC 9207). *P. aeruginosa* and *S. boydii* were subcultured on nutrient agar for 24 h at 37°C before any susceptibility testing was conducted. EPEC was subcultured on Dulbecco′s modified Eagle′s medium (DMEM) in a shaking incubator at 37°C until they developed an orange hue, signifying optimal growth (OD600‐0.6) [18]. Gentamicin‐ and amikacin‐resistant clinical isolates of *E coli*, *K. pneumoniae*, and *P. aeruginosa* were collected from the microbiology department of Rajshahi Medical College (RMC) Hospital. Resistance profiles were determined for each bacterium using standard antibiotic disks of gentamicin and amikacin.

### 2.3. Ethics Statement

The study was performed in accordance with the guidelines of the Institutional Animal, Medical Ethics and Biosecurity Committee of the Institute of Biological Sciences, University of Rajshahi, Rajshahi‐6205, Bangladesh. The approved experimental protocol was (memo No: 09(17)/320/IAMEBBC/IBSc).

### 2.4. Experimental Animal

For the biological activity study, Swiss Albino mice measuring 15–25 g and aged 3–4 weeks were obtained from the animal house, Katakhali, Rajshahi. Throughout the entire investigation, polypropylene cages were utilized to house the Swiss Albino mice. The environmental temperature was controlled and maintained at (24 ± 1°C), with a 12‐h light and 12‐h dark cycle. The humidity level was maintained at 60%–70%.

### 2.5. In Vitro Antimicrobial Tests

#### 2.5.1. Minimum Inhibitory Concentration (MIC) Determination

Resazurin dye incorporated standard broth microdilution method was conducted following the guidelines outlined by CLSI. Ninety‐six‐well microtiter plates with flat bottoms were used to assess the MIC. Antibiotics, nonantibiotics, and their combinations were dissolved in Mueller–Hinton broth (MHB). Two hundred microliter of MHB was added to Column 1, whereas only 100 *μ*L of MHB was added to Columns 2–10. For positive control, Column 11 was filled with 100 *μ*L of diluted inoculum, whereas Column 12 received 100 *μ*L of MHB to ensure sterility (negative control). Subsequently, MHB was transferred serially through a multichannel pipette from Columns 1 to 10, resulting in 100 *μ*L of agent in each well. Thus, through double serial dilutions, the concentrations in Columns 1–10 were as follows: oxytetracycline and its combinations (1024 *μ*g/mL to 2 *μ*g/mL), doxycycline and its combinations (64 *μ*g/mL to 0.03125 *μ*g/mL). Then, a 1:100 diluted bacterial inoculum was added to MHB. All wells comprising antibiotics, nonantibiotics, and their combinations, as well as positive and negative control wells, received 50 *μ*L of the OD600‐adjusted inoculum with an approximate concentration of 5 × 10^5^ CFU/mL. Bacterial inoculum was prepared and distributed to the wells within 15 min. Subsequently, 96‐well plates were covered with a lid and subjected to a 24‐h incubation period at 37°C. Fifty microliter of resazurin (0.015%) solution was added to each well, followed by an additional incubation period of 2–4 h. Columns that exhibited no alteration in color (with the blue resazurin color remaining unchanged) were taken as the MIC value after the completion of the incubation period. MIC values represent the concordant result of *n* = 3 independent biological replicates [19].

#### 2.5.2. Fractional Inhibitory Concentration Index (FICI) Determination

The FICI was determined to evaluate the synergism, no interaction, and antagonism between antibiotic and nonantibiotic combinations. FICI was calculated from the MIC values using the following formula such as FICI_Oxy+Sily_ = (MIC of Oxytetracycline in combination/MIC of Oxytetracycline alone) + (MIC of silybin in combination/MIC of silybin alone). The determined values were classified as synergism, no interaction, and antagonism based on the following criteria: FICI ≤ 0.5 determined as synergy, FICI > 4.0 determined as antagonism, and 0.5 < FICI ≤ 4 determined as no interaction [20].

### 2.6. Time‐Kill Kinetics Assay

Time‐kill kinetics was carried out for the EPEC. The concentration of the bacteria was first adjusted to a 0.5 McFarland standard, corresponding to an inoculum size of 1.0 × 10^6^ CFU/mL. Solutions containing the MIC concentration of gentamicin, Gen + Sily, and Gen + Dic were individually prepared and transferred to distinct test tubes containing sterile broth. The inoculum was added to each test tube, which was then incubated at 37°C for 24 h. Aliquots of 100 *μ*L were taken from each tube at time intervals of 0, 1, 3, 5, 7, 9, 12, and 24 h, which were then inoculated in a sterile petri dish containing nutrient agar. After 24 h of incubation at 37°C, colony counting was performed for each dish. A control test was performed without any drugs. The log value of colony‐forming unit (CFU/mL) was calculated, and a graph of log CFU/mL was plotted against time [21]. The procedure was performed three times to replicate the values.

### 2.7. In Vivo EPEC Mouse Model Study

Following a week of adjustment to the environment, all the mice were separated into five groups. Each group consisted of six mice. Two hundred and fifty microliter of cultured EPEC suspension (4.8–5 × 10^8^ CFU) was given orally to mice in each group except the control group with a gavage feeder at Day 1. The control group mice received only saline water treatment. Three treatment groups received different treatments intraperitoneally, where the gentamicin group received a dose (8 mg/kg of bw) [22] given twice a day for 3 consecutive days with a normal diet. For combination 1 group, the dose was gentamicin (8 mg/kg of bw) + silybin (150 mg/kg of bw) [23] and for the combination 2 group, the dose was: gentamicin (8 mg/kg of bw) + diclofenac (2.5 mg/kg of bw) [24], where doses were given twice a day for 3 consecutive days with a normal diet.

### 2.8. Monitoring Body Weight

Weight measurements were taken on the day of disease induction (Day 1) and subsequently on Days 2, 3, and 4 of the treatment periods using a digital weight machine with four‐digit precision.

### 2.9. Total White Blood Cell (WBC) Count

Total WBC count was determined for collected blood specimens using an automated veterinary hematology counter pocH‐100iV Diff (Sysmex–Roche), following the specified protocol [25].

### 2.10. Colitis, Edema, and Histopathological Analysis

To assess inflammation and edema, the weight of the colon was measured using a digital scale with four‐digit precision, whereas its length was measured using a ruler; photographs were also taken. Furthermore, to examine the protective effects of treatment groups against EPEC–induced diarrhea, a section of the colon was rinsed with sterile saline water, then immersed in 10% (v/v) buffered formaldehyde, and stored at room temperature for subsequent colonic sectioning. Colonic sections (5 *μ*m thick) were subjected to a staining process (hematoxylin & eosin) and examined under a fluorescence microscope [26].

### 2.11. Blood Culture

Blood specimens obtained in EDTA‐K2 tubes were incubated in the incubator to facilitate positive growth. Subsequently, blood cultures that tested positive underwent gram staining and were subcultured on Luria–Bertani (LB) agar and nutrient agar media [27].

### 2.12. In Silico Analysis

#### 2.12.1. Protein Preparation and Ligand Selection

Three‐dimensional crystal structures of the seven candidate bacterial targets, LpxA (PDB ID: 1LXA), BamA (PDB ID: 8ADG), AcrB (PDB ID: 4DX5), LolA (PDB ID: 7Z6W), MdfA (PDB ID: 4ZP0), FepA (PDB ID: 1FEP), and the *β*‐sliding clamp (PDB ID: 4K3S), originating from *E. coli*, were acquired from the Protein Data Bank [28]. Protein preparation was performed using Discovery Studio software v25.1.0.24284 (https://discover.3ds.com/discovery-studio-visualizer-download) to ensure data quality and consistency. Initially, protein structures underwent a cleaning procedure, eliminating heteroatoms and other extraneous elements. After this, energy minimizations were carried out on the protein structure utilizing GROMOS96 force field, in the SwissPDB Viewer software v4.1.0 (accessible at https://spdbv.unil.ch/disclaim.html) [29]. Silybin and diclofenac were selected as ligands for docking investigation.

#### 2.12.2. Molecular Docking Study

Between several bacterial target proteins and ligands such as silybin and diclofenac, a virtual screening molecular docking study was performed using PyRx software v0.8 (accessible at https://sourceforge.net/projects/pyrx/) with Vina Wizard [30]. Initially, the protein structures were loaded into PyRx software and then converted into macromolecules; whereas the ligands were loaded, energy was minimized using the mmff94 force field combined with the steepest descent algorithm and converted into PDBQT format. The final docking calculations were conducted using PyRx, and the protein–ligand complex was selected based on the lowest binding energy. Nonbonding interactions and poses were visualized using Discovery Studio software [31].

#### 2.12.3. MD Simulation

MD simulations were performed for 250 ns using the GROMACS 2024.1 [32] software package. The protein topology was generated using the CHARMM36 all‐atom force field (charmm36‐jul2022.ff) [33], whereas the ligand topologies were constructed via the CGenFF server (https://cgenff.com/) [34]. Specifically, the 4K3S + diclofenac system was contained within a box of dimensions 4.391 × 7.122 × 7.713 nm, resulting in an approximate system diameter of 8.528 nm. The 7Z6W + silybin system was placed in a box measuring 4.122 × 4.354 × 5.820 nm, with a system diameter of 6.423 nm. Solvation was carried out with the TIP3P water model, followed by the addition of Na^+^ and Cl^−^ counter‐ions to neutralize the system at an ionic strength of 0.15 M [35]. Before conducting MD simulations, energy minimization was performed on each system using the steepest descent algorithm for up to 50,000 steps until the maximum force fell below 1000 kJ mol^−1^ nm^−1^. Following minimization, each protein–ligand complex was subjected to a two‐step equilibration protocol for 100 ps in both canonical (NVT) and isothermal‐isobaric (NPT) ensembles under position restraints on heavy atoms. The system temperature was maintained at 300 K using the velocity‐rescaling (V‐rescale) thermostat [36], and a pressure of 1 bar was maintained during NPT equilibration using the Berendsen barostat [37] with isotropic cell scaling. Long‐range electrostatic interactions were calculated via the Particle Mesh Ewald (PME) method [38] with a Fourier grid spacing of 0.16 nm and a cutoff of 1.2 nm. Short‐range van der Waals interactions were smoothly switched off between 1.0 nm and 1.2 nm in accordance with CHARMM force field parameters. All hydrogen‐containing bonds were constrained using the LINCS algorithm [39]. Following equilibration, unrestrained 250 ns production runs were conducted in triplicate. Trajectories were subsequently analyzed using standard GROMACS utilities.

### 2.13. Molecular Mechanics/Poisson–Boltzmann Surface Area (MM/PBSA)

To assess the binding affinity between the protein and ligand, binding free energy calculations were performed using the MM/PBSA approach [40]. These computations were carried out with the gmx_MMPBSA tool (Version 1.6.4) [41], which represents an adaptation derived from the AMBER MMPBSA.py framework [42]. For these analyses, 10 representative frames capturing snapshots of the protein–ligand complex were sampled from the MD simulation trajectory.

The binding free energy (*Δ* *G*_*b* *i* *n* *d*_) is calculated as the difference between the free energy of the complex and the individual free energies of the receptor and ligand:

ΔGbind=Gcomplex−Greceptor+Gligand.

Each free energy term (G) in the equation is determined using the following expression:

Gx=EMM+Gsolv−TΔS,

where *E*_*M* *M*_ represents the molecular mechanics energy in the gas phase, *G*_*s* *o* *l* *v*_ is the solvation free energy, and T*Δ*S is the entropic contribution. The calculation of the entropic term is computationally intensive and was therefore omitted in this study, a common practice in MM/PBSA analyses.

The molecular mechanics energy (*E*_*M* *M*_) is the sum of both bonded and nonbonded interactions:

EMM=Ebonded+Enon−bonded=Ebond+Eangle+Edihedral+EvdW+Eelec.

Since the simulation does not involve the formation or breaking of covalent bonds, the contribution from the bonded energy terms (*Δ* *E*_*b* *o* *n* *d* *e* *d*_) cancels out when calculating the final binding free energy. Therefore, the change in molecular mechanics energy (*Δ* *E*_*M* *M*_) is composed of the changes in van der Waals (*Δ*E_vdW) and electrostatic (*Δ* *E*_*e* *l* *e* *c*_) energies.

The solvation free energy (*G*_*s* *o* *l* *v*_) is composed of two components: the polar (*G*_*p* *o* *l* *a* *r*_) and nonpolar (*G*_*n* *o* *n* *p* *o* *l* *a* *r*_) contributions:

Gsolv=Gpolar+Gnonpolar.

In this study, the polar solvation energy (*G*_*p* *o* *l* *a* *r*_) was calculated by solving the Poisson–Boltzmann (PB) equation. The nonpolar contribution to the solvation free energy (*G*_*n* *o* *n* *p* *o* *l* *a* *r*_) was determined based on the solvent accessible surface area (SASA), calculated using the Linear Combination of Pairwise Overlaps (LCPO) algorithm [43]. The final binding free energy is thus calculated as the sum of the changes in the gas‐phase and solvation energy components:

ΔGbind=ΔEMM+ΔGsolv=ΔEvdW+ΔEelec+ΔGpolar+ΔGnonpolar.

### 2.14. Statistical Analysis

Statistical analysis was performed using GraphPad Prism software (v10.1.2). The *p* values were calculated at a 95% confidence interval (CI) using one‐way ANOVA, and multiple comparison tests were performed using Tukey′s multiple comparisons test. Asterisks denote significant differences among groups, where  ^∗^ indicates *p* < 0.05,  ^∗∗^ indicates *p* < 0.01,  ^∗∗∗^ indicates *p* < 0.001, and  ^∗∗∗∗^ indicates *p* < 0.0001.

## 3. Results

### 3.1. MICs of Drug Combinations and Their FICI Interpretations

The MIC values of antibiotics, nonantibiotics, and their combinations are outlined in Table 1 for the laboratory strains of *P. aeruginosa*, EPEC, and *S. boydii*, and in Table 2 for the gentamicin‐ and amikacin‐resistant clinical isolates of *E. coli*, *K. pneumoniae*, and *P. aeruginosa*, respectively. Drug combinations that showed synergism and significant MIC fold reduction are marked with  ^∗^, as interpreted by the FICI values (Table S1 and Table S2). For pathogenic bacteria, silybin and diclofenac exhibited noteworthy MICs against *E. coli* and *S. boydii*, but no significant MIC was observed for *P. aeruginosa*. Additionally, combination drugs were also ineffective in reducing the MIC against *P. aeruginosa*. In the case of EPEC, Doxy + Dic and Gen + Dic showed notable four‐fold reductions in MIC. In *S. boydii*, both Oxy + Dic and Ery + Dic exhibited three‐fold reductions in MIC. Additionally, five‐fold reductions in MIC were observed for both the Gen + Sily and Gen + Dic combinations.

**Table 1:** Represents MIC in *μ*g/mL of antibiotics, nonantibiotics, and their combinations against susceptible laboratory reference strains.

Drugs	*P. aeruginosa* (ATCC 27853)	*E. coli* (EPEC)	*S. boydii* (ATCC 9207)
Sily	512	128	128
Dic	512	64	256
Oxy	32	2	2
Oxy + Sily	32	2	2
Oxy + Dic	32	2	0.25^a^ (threefold)
Doxy	16	32	2
Doxy + Sily	32	8^a^ (twofold)	1
Doxy + Dic	16	2^a^ (fourfold)	0.5^a^ (twofold)
Chl	64	64	4
Chl + Sily	64	32	4
Chl + Dic	32	16^a^ (twofold)	4
Cip	0.5	4	0.0625
Cip + Sily	0.5	2	0.125
Cip + Dic	0.5	1^a^ (twofold)	0.0625
Pen G	512	64	32
Pen G + Sily	512	128	16
Pen G + Dic	512	128	16
Ery	64	4	8
Ery + Sily	64	4	4
Ery + Dic	32	8	1^a^ (threefold)
Azi	128	256	4
Azi + Sily	64	256	8
Azi + Dic	128	256	4
Gen	2	4	16
Gen + Sily	2	1^a^ (twofold)	0.5^a^ (fivefold)
Gen + Dic	2	0.25^a^ (fourfold)	0.5^a^ (fivefold)

Abbreviations: Azi, azithromycin; Chl, chloramphenicol; Cip, ciprofloxacin; Dic, diclofenac; Doxy, doxycycline; Ery, erythromycin; Gen, gentamicin; Oxy, oxytetracycline; Pen G, penicillin G; Sily, silybin.

^a^Synergism between antibiotics and nonantibiotics according to calculated FICI values. In all cases, no interreplicate variance was observed.

**Table 2:** Represents MIC in *μ*g/mL of gentamicin, amikacin and their combinations with silybin and diclofenac against gentamicin‐ and amikacin‐resistant bacteria.

Drugs	*E. coli*	*K. pneumoniae*	*P. aeruginosa*
Sily	8	16	256
Dic	16	16	256
Gen	32	8	1024
Gen + Sily	2^a^ (fourfold)	2^a^ (twofold)	512
Gen + Dic	2^a^ (fourfold)	2^a^ (twofold)	512
Ami	16	32	1024
Ami + Sily	8	16	512
Ami + Dic	8	16	512

Abbreviations: Ami, amikacin; Dic, diclofenac; Gen, gentamicin; Sily, silybin.

^a^Synergism between antibiotics and nonantibiotics according to calculated FICI values. In all cases, no inter‐replicate variance was observed.

Gen + Sily and Gen + Dic were the most promising synergistic combinations for the laboratory strains of EPEC and *S. boydii*. Their efficacy against gentamicin‐ and amikacin‐resistant clinical isolates was evaluated in Table 2 and found to be clinically significant. Although a similar pattern was observed in resistant *P. aeruginosa*, notable MIC reductions were seen in both *E. coli* and *K. pneumoniae*. Gentamicin combinations reduced MICs fourfold and twofold against resistant *E. coli* and *K. pneumoniae*, respectively, whereas only minimal MIC reduction was observed with amikacin, another aminoglycoside‐class antibiotic. Beyond fold‐change, the clinical significance of these reductions can be interpreted using CLSI breakpoints. According to the breakpoints in CLSI M100 32nd or 33rd edition, gentamicin susceptibility is defined as MIC ≤2 *μ*g/mL, with resistance at ≥ 8 *μ*g/mL. By this standard, the resistant *E. coli* isolate in Table 2, (gentamicin MIC 32 *μ*g/mL, above the resistant breakpoint) was shifted into the susceptible range by both Gen + Sily and Gen + Dic (MIC 2 *μ*g/mL), and the resistant *K. pneumoniae* isolate (gentamicin MIC 8 *μ*g/mL, at the resistant breakpoint) was shifted well within the susceptible range by both combinations (MIC 2 *μ*g/mL). This breakpoint‐crossing effect, rather than fold‐change alone, can restore gentamicin as a treatment option for resistant isolates in practice [44].

### 3.2. Time‐Kill Kinetics Analysis

Time‐kill assays were performed to evaluate the viable cell count reduction capability of gentamicin and its combination over 24 h, which is depicted in Figure 1a. The control group exhibited continuous bacterial proliferation, where at 0‐h, colony count was 5.72 log_10_, which ultimately reached a level of 7.31 log_10_ CFU/mL at 24 h. Gentamicin monotherapy initially reduced bacterial counts from 5.70 log_10_ CFU/mL to 2.52 log_10_ CFU/mL by 5 h, which corresponded to a ≥3 log_10_ CFU/mL reduction from baseline. However, a similar pattern of viable colony reduction up to 5 h was also seen for both of the combinations, Gen + Sily and Gen + Dic. After 5 h, bacterial regrowth was observed in the gentamicin group, where the count rose to 4.15 log_10_ CFU/mL at 24 h. In contrast, both combinations demonstrated sustained antibacterial activity. For Gen + Sily, bacterial counts existed at 5.71 log_10_ CFU/mL, which completely returned to an undetectable level by 7 h. Gen + Dic also produced an undetectable colony count by 9 h. Compared with gentamicin monotherapy, this combination demonstrated a ≥2 log_10_ CFU/mL greater reduction at corresponding time points (7–9 h), which remained for the rest of the 24 h. According to established time‐kill interpretation criteria, both combinations exhibited synergistic interactions (≥2 log_10_ CFU/mL greater killing than the most active single agent) and bactericidal activity, whereas gentamicin monotherapy showed only a transient bactericidal effect followed by regrowth.

**Figure 1:**
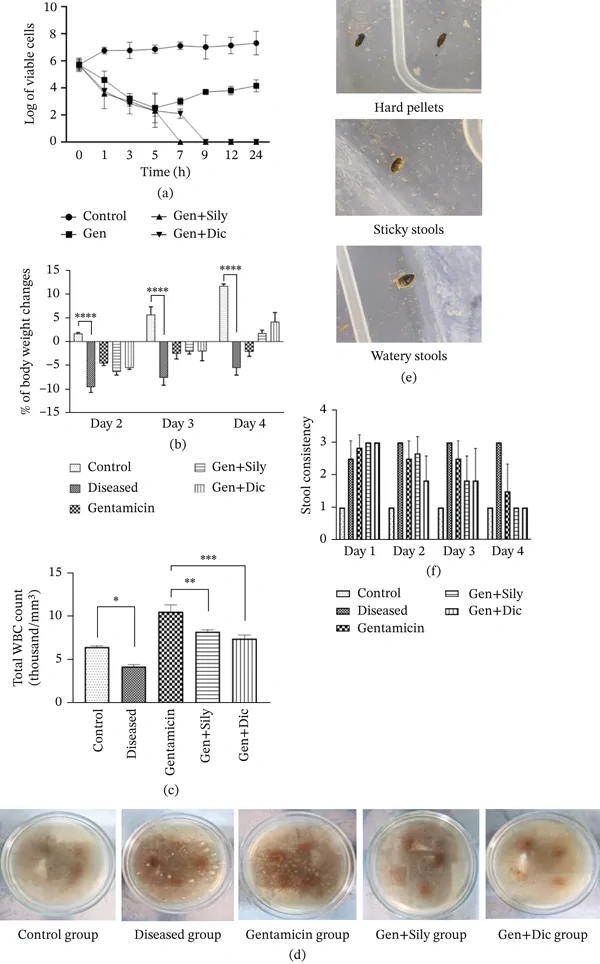
(a) Log of viable cell counts at MIC concentrations of gentamicin and its combinations. For control, diseased, and different treatment groups: (b) Graph illustrating the percentage of body weight changes. (c) Graph illustrating total white blood cell (WBC) count. (d) Blood culture test. (e) Visual stool scoring criteria. (f) Graph illustrating stool consistency from Day 1 to Day 4. In this figure, asterisks denote statistically significant differences among groups:  ^∗^ indicates *p* < 0.05,  ^∗∗^ indicates *p* < 0.01,  ^∗∗∗^ indicates *p* < 0.001, and  ^∗∗∗∗^ indicates *p* < 0.0001.

### 3.3. In Vivo EPEC Mouse Model Study

#### 3.3.1. Gentamicin and Its Combination Effect on Body Weight

Body weight was measured on the day of disease onset (Day 1) and then on consecutive treatment days (Days 2, 3, and 4), represented as the percentage of body weight changes in Figure 1b. The control group exhibited a gradual increase in body weight, whereas the diseased group demonstrated a significant decline. Notable statistical differences in body weight changes were observed between the control and diseased groups on Day 2 (*p* < 0.0001), Day 3 (*p* < 0.0001), and Day 4 (*p* < 0.0001). Additionally, the gentamicin, Gen + Sily, and Gen + Dic groups displayed a decrease in body weight on Day 2, followed by an increase on Days 3 and 4.

### 3.4. Gentamicin and Its Combination Effect on Total WBC Count

Total WBC count, demonstrated in Figure 1c, showed a decrease in WBC count in the diseased group (4.3 ± 0.208 thousand/mm^3^), compared with the control group (6.51 ± 0.150 thousand/mm^3^), which was statistically significant (*p* = 0.0106 < 0.05). The reduced WBC count suggested continuous consumption of WBCs produced by the bone marrow at the site of infection. Gentamicin maintained the WBC level at 10.56 ± 0.808 thousand/mm^3,^ which was significantly higher than the Gen + Sily group, 8.26 ± 0.23 thousand/mm^3^ (*p* = 0.0076 < 0.01), and the Gen + Dic group, 7.5 ± 0.4 thousand/mm^3^ (*p* = 0.0003 < 0.001).

### 3.5. Gentamicin and Its Combination Effect on Blood Culture

Blood specimens were subjected to blood culture to detect bloodstream infections. The blood culture was conducted using LB agar and nutrient agar media. The nutrient agar culture revealed no bacterial growth across the five groups tested, whereas the LB agar demonstrated substantial growth of bacterial colonies in the diseased group and low growth in the gentamicin group, as shown in Figure 1d. In the case of the diseased group, a higher bacterial colony count was observed, indicating the presence of pathogens in the bloodstream and yielding a positive blood culture result. Conversely, no visible bacterial growth was observed in blood cultures from the Gen + Sily and Gen + Dic groups.

### 3.6. Gentamicin and Its Combination Effect on Stool Scoring

Stool scoring was conducted from the day of disease induction (Day 1), which continued to the whole treatment period (Days 2–4) that is illustrated in Figure 1f, following the criteria in Figure 1e such as hard pellets were scored as 1, sticky stools adhering to the cage or microtube wall were scored as 2, watery stools with or without mucus were scored as 3, and bloody stool was scored as 4. On Day 1, a notable difference in stool scoring was observed between the control group and the diseased group, which became more pronounced on Days 2–4 as diarrhea persisted in the diseased group. By Day 4, the stool scoring for the Gen + Sily and Gen + Dic groups closely resembled that of normal mice, with a difference observed in the gentamicin group.

### 3.7. Gentamicin and Its Combination Effect on Colitis and Edema

Colon shortening is a common symptom of colitis [26]. As depicted in Figure 2a,b, the colon length was reduced in the diseased group (6.7 ± 0.54 cm) compared with the control group (10.2 ± 1.52 cm). The colon length measured for Gen + Sily and Gen + Dic groups was 10.5 ± 0.58 cm and 10.9 ± 0.41 cm, whereas the length was 9.6 ± 0.69 cm for the gentamicin group. It is recognized that edema is also a characteristic symptom of colitis. The extent of edema could be indirectly assessed by the colon weight/length (mg/cm) ratio, with a higher ratio indicating more severe edema. As illustrated in Figure 2c, the ratio increased markedly in the diseased group (88.41 ± 2.54 mg/cm) (*p* < 0.0001) compared with that of the control group (73.63 ± 1.46 mg/cm). Colon weight/length ratio was identified as 118.56 ± 1.70 mg/cm in the gentamicin group, 72.22 ± 1.34 mg/cm in the Gen + Sily group, and 87.39 ± 1.23 mg/cm in the Gen + Dic group. Severe edema was detected in the gentamicin group (*p* < 0.0001) compared with both the Gen + Sily group and Gen + Dic group.

**Figure 2:**
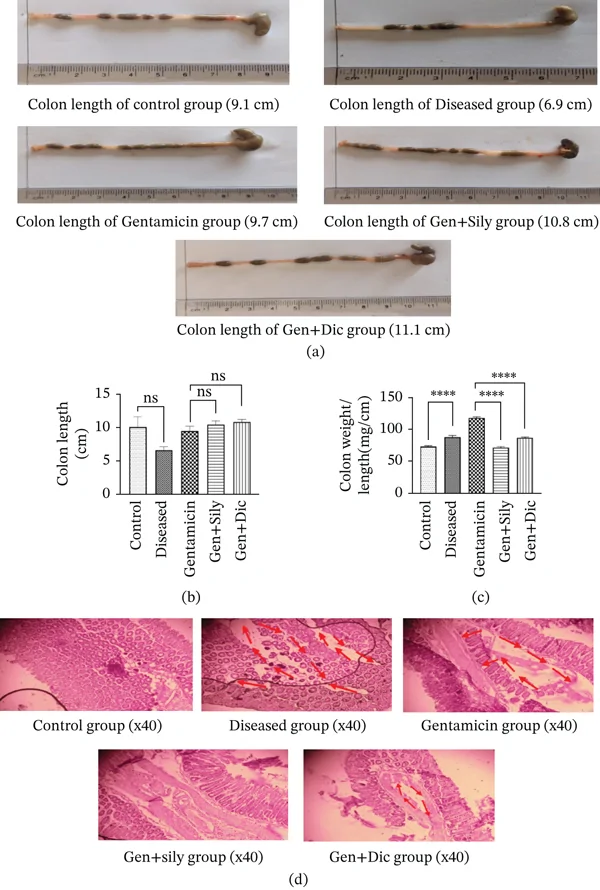
(a) Morphological observation of the colon and length measurement. (b) Graph illustrating colon length. (c) Graph illustrating colon weight/length ratio. (d) Histopathological observation of the colon. In this figure, asterisks denote statistically significant differences among groups, with  ^∗∗∗∗^ indicating *p* < 0.0001.

### 3.8. Gentamicin and Its Combination Effect on Histopathology

The colonic sections from different groups illustrated in Figure 2d underwent H&E staining and were subsequently examined under a fluorescence microscope to assess the protective effects of the treatment groups. Colonic sections from the diseased group exhibited damage to the mucosal lining and extensive infiltration of inflammatory cells compared with the control mice. In contrast, the colonic tissue samples from mice treated with Gen + Sily exhibited nearly normal structure. Gen + Dic showed lower damage compared with the colonic section of mice that received gentamicin alone. The colonic structure from the Gen + Sily group was almost as intact as that of the control group mice, with healthier colonic mucosal epithelium, well‐preserved crypts, and reduced inflammatory reactions. These findings indicated that both Gen + Sily and Gen + Dic combinations effectively mitigate colitis triggered by EPEC and preserve colonic mucosal tissue, outperforming the effects of gentamicin monotherapy.

### 3.9. In Silico Study

#### 3.9.1. Virtual Screening Molecular Docking Study

After conducting an extensive literature review on the potential targets of silybin and diclofenac, it was hypothesized that their targets are involved in altering membrane permeability or inhibiting efflux pumps, thereby increasing intracellular antibiotic accumulation and ultimately enhancing their activity as antibiotic adjuvants. Based on this hypothesis, several protein targets were selected whose modulation is expected to increase antibiotic concentrations within bacterial cells. In addition, several studies have reported that NSAIDs directly inhibit the *β*‐sliding clamp protein, thereby disrupting DNA replication [16, 45]. To evaluate these hypotheses, proteins associated with membrane permeability and efflux mechanisms were selected as potential targets for silybin. For diclofenac, these proteins, along with the *β*‐sliding clamp protein, were selected based on its reported role as a molecular target of NSAIDs. Following this, a virtual screening molecular docking study was performed using bacterial protein targets LpxA (1LXA), BamA (8ADG), AcrB (4DX5), LolA (7Z6W), MdfA (4ZP0), FepA (1FEP), and the *β*‐sliding clamp (4K3S) [45–47]. From the virtual screening results, the current study selected LolA and the *β*‐sliding clamp protein for further GROMACS simulation analysis.

Based on the literature on the Lol system, the current study targeted the periplasmic chaperone LolA, which contains a hydrophobic lipoprotein‐binding cavity composed of the following amino acid residues: Leu10, Val13, Phe16, Leu39, Val41, Arg43, Phe47, Trp49, Leu59, Leu66, Phe68, Ala84, Thr85, Thr88, Pro89, Phe90, Leu92, Ile93, Leu115, Leu124, Ile129, Ile137, Phe140, Ala142, Ser150, Tyr152, Leu154 [15]. Its functional activity centers on a ~1400 Å^3^ central hydrophobic cavity formed by an 11‐stranded antiparallel *β*‐barrel capped by a dynamic *α*‐helical lid. Structural studies identify Phe47 and Trp49 as key aromatic residues stabilizing the cavity. Access to the cavity is regulated by Arg43, which stabilizes the closed conformation via intramolecular hydrogen bonding and undergoes displacement during substrate loading [48].

Another promising strategy for developing effective antibiotics is inhibiting bacterial DNA replication, as exemplified by fluoroquinolones, which target DNA gyrase and topoisomerase IV [49]. The *β*‐sliding clamp, a subunit of DNA polymerase III, is a homodimeric toroidal protein that encircles double‐stranded DNA, serving as an essential processivity factor for the DNA polymerase III complex. DNA transits the central channel at a 22° tilt to facilitate factor switching, stabilized by conserved residues Arg24, Gln149, Arg73, and Arg80. Furthermore, Tyr153 and Tyr154 facilitate loading by stacking with template ssDNA. Targeting these clefts offers a dual therapeutic advantage by arresting replication fork progression and blocking DNA repair pathways, thereby inhibiting bacterial survival and the emergence of antibiotic resistance [50, 51].

Molecular docking analysis of protein–ligand complexes with their interacting residues, interaction type, and interaction distance is outlined in Table S3. In the case of the 4K3S protein (*β*‐sliding clamp), diclofenac in Figure 3b exhibited higher thermodynamic stability (−6.4 kcal/mol) than the standard drug (PubChem CID73010404) [17] (−5.8 kcal/mol) in Figure 3a, utilizing a high‐specificity halogen bond with Asn295 and dual‐mode stabilization through *π π* ‐cation and *π π* ‐alkyl interactions with Arg282. By efficiently occupying the conserved hydrophobic cleft, diclofenac probably sterically hinders the attachment of partner protein clamp‐binding motifs, thereby halting replication fork progression and compromising essential DNA repair pathways. For the 7Z6W protein (LolA), silybin exhibited a binding affinity of −10.4 kcal/mol in Figure 3e compared with the standard inhibitor MAC13243 in Figure 3c (PubChem CID2806781) [52] (−8.7 kcal/mol) and the native lipoprotein (−7.2 kcal/mol) in Figure 3d. This robust stability is anchored by a network of four conventional hydrogen bonds with Ser14, Lys42, Arg43, and Tyr152, alongside critical hydrophobic contacts with Phe47 and Trp49.

**Figure 3:**
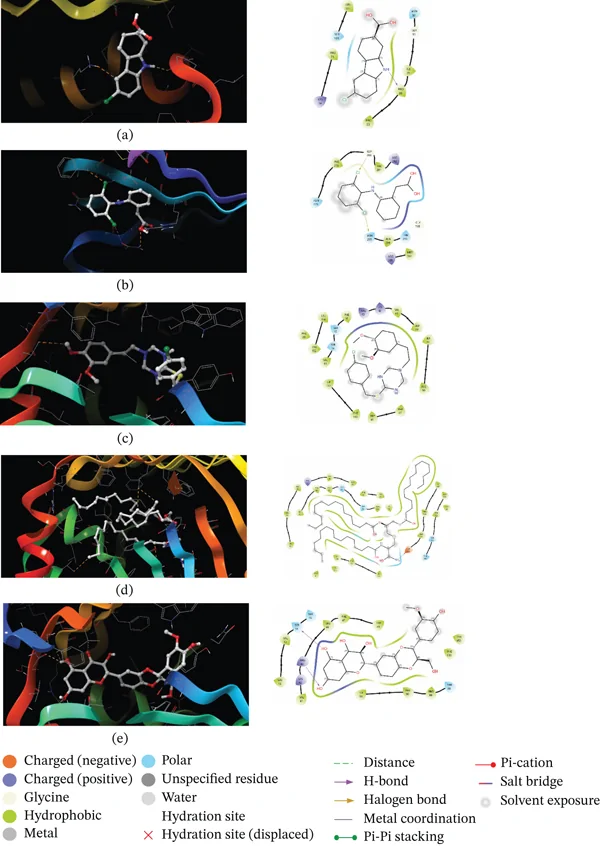
Molecular docking orientations and 2D residual interaction maps. (a) *E. coli β*‐sliding clamp (PDB ID: 4K3S) in complex with the reference standard ligand (CID73010404). (b) 4K3S in complex with drug candidate (diclofenac). (c) *E. coli* LolA (PDB ID: 7Z6W) transporter in complex with the reference standard ligand (MAC13243). (d) 7Z6W in complex with the natural lipoprotein cargo. (e) 7Z6W in complex with the drug candidate (silybin).

### 3.10. MD Simulation

#### 3.10.1. Root Mean Square Deviation (RMSD)

The RMSD trajectories across the 250 ns production run reveal distinct patterns of structural stability and equilibration behavior among the simulated systems. In the case of the 4K3S protein (*β*‐sliding clamp) in Figure 4a, the 4K3S + standard complex equilibrated quickly and settled into a stable plateau around 0.25 nm, pointing to considerable structural stabilization compared with the more dynamic apo state. The 4K3S + diclofenac complex exhibited greater mobility, with RMSD values fluctuating between 0.25 and 0.40 nm, though these deviations remained notably lower than the prominent peaks seen in the apo form, suggesting that diclofenac confers a moderate level of conformational constraint. For the 7Z6W protein (LolA) in Figure 4b, substantial intrinsic flexibility was evident in both the apo and 7Z6W + standard, where RMSD values routinely surpassed 0.6 nm. Interestingly, 7Z6W + silybin appears to promote a gradual structural transition toward enhanced stability, with RMSD values progressively converging below 0.4 nm after roughly 175 ns. Although the 7Z6W + lipoprotein stabilized earlier during the simulation, the delayed convergence observed with the silybin complex is indicative of a slower conformational adaptation into a more stable bound state.

**Figure 4:**
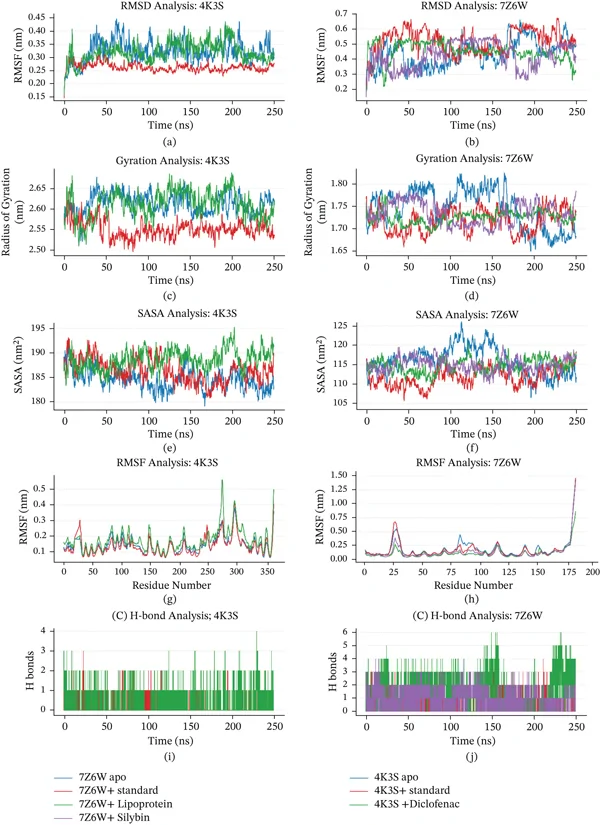
Comparative molecular dynamics trajectories over 250 ns production simulations. (a) Backbone RMSD for 4K3S systems. (b) Backbone RMSD for 7Z6W systems. (c) Radius of gyration (Rg) for 4K3S systems. (d) Radius of gyration (Rg) for 7Z6W systems. (e) SASA for 4K3S systems. (f) SASA for 7Z6W systems. (g) Per‐residue RMSF for 4K3S systems. (h) Per‐residue RMSF for 7Z6W systems. (i) Intermolecular hydrogen bond count for 4K3S complexes. (j) Intermolecular hydrogen bond count for 7Z6W complexes.

#### 3.10.2. Radius of Gyration (Rg)

The overall compactness and structural coherence of the simulated systems were assessed through Rg analysis across the 250 ns production phase. For the 4K3S protein (*β*‐sliding clamp) in Figure 4c, the apo form exhibited a relatively expanded and highly variable profile, whereas the 4K3S + standard complex appeared markedly more compact, sustaining a consistently lower Rg throughout the simulation. The 4K3S + diclofenac complex displayed greater conformational expansion and intermittent fluctuations compared with the standard complex, indicating a less constrained or more structurally flexible state upon binding of this ligand. For the 7Z6W protein (LolA) in Figure 4d, the apo form showed the most pronounced structural expansion and instability, marked by substantial oscillations in Rg values. 7Z6W + standard and 7Z6W + lipoprotein appeared to preserve the protein in a more compact and stable conformation relative to the apo state. Interestingly, the 7Z6W + silybin demonstrated an overall trend toward enhanced structural compactness compared with the apo form, though fluctuations in the later stages of the trajectory suggest that the protein may continue to explore a wider conformational ensemble, potentially reflecting a more dynamic or partially restrained binding interaction.

#### 3.10.3. SASA

The SASA trajectories reveal that ligand binding produces characteristic patterns of solvent exclusion and surface exposure. In the 4K3S protein (*β*‐sliding clamp) in Figure 4e, 4K3S + diclofenac appeared to sustain a relatively elevated SASA (∼188–194 nm^2^) compared with both the apo and 4K3S + standard complex, with a notable trend toward increased solvent exposure during the final 100 ns of the simulation. This observation may suggest that diclofenac binding favors a more solvent accessible or partially open conformational state. For the 7Z6W protein (LolA) in Figure 4f, the apo form exhibited considerable fluctuations in surface accessibility, including a distinct peak surpassing 125 nm^2^ between 100 and 150 ns, likely reflecting transient opening of the internal binding cavity. Binding of the standard and native lipoprotein cargo effectively minimized these expansions, preserving a more compact and solvent shielded surface topology. Conversely, the 7Z6W + silybin complex displays an intermediate SASA profile, showing greater surface accessibility than the lipoprotein‐bound system throughout much of the trajectory, while generally remaining below the maximal exposure levels observed in the apo state.

#### 3.10.4. Root mean square fluctuation (RMSF)

RMSF analysis over the 250 ns trajectories demonstrated that ligand binding distinctly influences regional backbone flexibility in a context‐dependent manner. In the 4K3S protein (*β*‐sliding clamp) in Figure 4g, the 4K3S + standard complex displays widespread stabilization of the protein backbone, whereas the 4K3S + diclofenac complex retained considerably greater mobility in functionally relevant loop regions, especially around residues 270–280, where fluctuation peaks surpass 0.5 nm. This region‐specific flexibility implies that diclofenac may permit a more dynamic and conformationally versatile clamp interface relative to the more constrained standard complex. Likewise, 7Z6W protein (LolA) in Figure 4h, the native lipoprotein cargo produces pronounced dampening of flexibility across the protein architecture, notably stabilizing the *α*‐helical lid domain (residues 25–35). Conversely, 7Z6W + silybin complex appeared to preserve heightened fluctuations within this lid region while inducing substantial mobility at the C‐terminal tail (residues 185–192), potentially reflecting a binding orientation that favors a more open or dynamically accessible lid conformation.

#### 3.10.5. Hydrogen Bond

The protein–ligand hydrogen bond trajectories across the 250 ns production run indicate that the test ligands form distinctive polar interaction networks within their respective binding sites. In the 4K3S protein (*β*‐sliding clamp) in Figure 4i, 4K3S + diclofenac exhibited a more extensively populated and sustained hydrogen bond profile relative to the 4K3S + standard complex, consistently maintaining one to two concurrent interactions with occasional increases to four bonds, which may facilitate more stable polar engagement at the binding interface. For the 7Z6W protein (LolA) in Figure 4j, the native lipoprotein cargo established the most comprehensive and temporally stable hydrogen bond network, regularly sustaining between four and six simultaneous bonds throughout the trajectory. By comparison, the 7Z6W + silybin complex displayed a more intermittent and less densely populated interaction profile, typically fluctuating between zero and two hydrogen bonds. This reduced occupancy and bond count relative to the lipoprotein cargo suggests that silybin binding may operate through a more dynamic engagement mechanism or an alternative arrangement of polar contacts within the transporter‐binding pocket.

### 3.11. MM/PBSA

The MM/PBSA analysis reveals distinct binding energetics across the protein–ligand complexes. For the 4K3S sliding clamp, 4K3S + diclofenac exhibits substantially stronger binding (−24.42 kcal/mol) compared with the 4K3S + standard (−8.17 kcal/mol) **(**Table 3), driven primarily by enhanced van der Waals interactions (−27.03 vs. −13.83 kcal/mol). For the 7Z6W protein (LolA), the native lipoprotein demonstrates the most favorable binding affinity (−41.91 kcal/mol), characterized by exceptionally strong van der Waals contributions (−89.49 kcal/mol). 7Z6W + Silybin shows intermediate binding strength (−28.88 kcal/mol), surpassing the 7Z6W + standard (−21.99 kcal/mol) but remaining weaker than the natural substrate. Across all complexes, van der Waals interactions constitute the primary driving force for binding, whereas polar solvation penalties partially offset electrostatic contributions. These results indicate that both test compounds achieve energetically competitive binding within their respective targets.

**Table 3:** MM/PBSA binding free energy components for protein–ligand complexes.

Energy component	4K3S + standard	4K3S + diclofenac	7Z6W + standard	7Z6W + lipoprotein	7Z6W + silybin
Van der Waals energy (kcal/mol)	−13.83 ± 1.42	−27.03 ± 3.22	−40.41 ± 3.17	−89.49 ± 3.65	−44.41 ± 1.60
Electrostatic energy (kcal/mol)	1.98 ± 2.81	0.20 ± 3.64	−90.16 ± 8.92	−2.78 ± 3.63	−11.59 ± 5.23
Polar solvation energy (kcal/mol)	4.86 ± 2.42	6.14 ± 1.78	113.36 ± 7.26	63.30 ± 5.07	33.22 ± 2.89
Nonpolar solvation energy (kcal/mol)	−1.19 ± 0.23	−3.73 ± 0.14	−4.81 ± 0.24	−12.95 ± 0.61	−6.10 ± 0.14
***Δ*G_bind (kcal/mol)**	−8.17 ± 1.58	−24.42 ± 3.03	−21.99 ± 3.18	−41.91 ± 3.57	−28.88 ± 2.88

*Note:* The bolded values indicate the calculated *Δ*G_bind (kcal/mol) values for the ligand‐bound complexes that exhibited more favorable (more negative) MM/PBSA binding free energies relative to their corresponding standard complexes, with the 7Z6W–lipoprotein complex showing the most favorable predicted binding free energy.

## 4. Discussion

To address the rising AMR challenge, the current study investigated the potential of the established drugs silybin and diclofenac as antibiotic adjuvants. Previously, the synergistic potential of antibiotic adjuvants has been reported [53], with silybin and diclofenac also being reported as antibiotic adjuvants in several studies. In the case of silybin, one study demonstrated the effectiveness of ampicillin–silybin and gentamicin–silybin combinations against oral bacteria, whereas another study reported that combinations of ampicillin and oxacillin were effective against methicillin‐resistant *Staphylococcus aureus* (MRSA) [54, 55]. In vitro and in silico analyses further demonstrated the inhibition of staphyloxanthin production and biofilm formation in *S. aureus*, ultimately potentiating ciprofloxacin activity [56]. Regarding the potential target of silybin, one study demonstrated the inhibition of efflux pump norA and qacA/B expression in MRSA [57, 58]. In the case of diclofenac, previous studies have reported its antibacterial activity against a broad range of susceptible and clinical strains of *S. aureus*, *Listeria monocytogenes*, *E. coli*, and *Mycobacterium tuberculosis* through inhibition of DNA synthesis [59]. Moreover, diclofenac enhanced the activity of oxytetracycline against MRSA and inhibited MRSA‐associated biofilm formation through the downregulation of *β*‐lactam resistance gene expression [60, 61]. Diclofenac also exerted an antivirulence effect that restores colistin susceptibility in MDR *A. baumannii* [24].

Similar to previous investigations, the current study also identified synergistic combinations of silybin and diclofenac with antibiotics from different pharmacological classes. Among these combinations, both silybin and diclofenac demonstrated significant synergistic interactions with gentamicin against laboratory strains of EPEC and *S. boydii*, as confirmed by MIC determination and checkerboard analysis (FICI). However, as different authors use varying FICI thresholds to define synergism, and values close to the interpretive threshold may be difficult to classify unequivocally, synergistic interactions should be further validated using time‐kill kinetics [62]. The time‐kill kinetic assays validated the synergistic effects of gentamicin in combination with silybin and diclofenac, clearly demonstrating the enhanced efficacy of these combinations compared with gentamicin monotherapy. Both gentamicin‐ and amikacin‐resistant isolates highlighted the specificity of the Gen + Sily and Gen + Dic combinations, as no synergism was observed with amikacin despite belonging to the same aminoglycoside class. However, despite the combinations demonstrating efficacy against pathogenic bacteria, they remained ineffective against *P. aeruginosa*. Although *P. aeruginosa*, *E. coli*, and *K. pneumoniae* are all gram‐negative bacteria, they differ in their cell envelope structure, membrane permeability, and antibiotic resistance mechanisms. *P. aeruginosa* is estimated to be 12–100 times less permeable than *E. coli* [63]. This difference may be attributed to the distinct major porins expressed by the two bacteria, namely, OprF in *P. aeruginosa* and OmpA, OmpF, and OmpC in *E. coli*. It was estimated that, of the approximately 200,000 OprF molecules, only 400 remain in the open‐conformer state. The exclusion limit of OmpF is approximately 600 Da; however, that of OprF is markedly lower (∼200 Da), leading to its designation as a slow porin [64]. In addition to this intrinsic impermeability, the efflux pump MexXY system is the key determinant of aminoglycoside resistance in *P. aeruginosa*. MexXY is significantly upregulated, conferring natural resistance to aminoglycosides [65]. Aminoglycoside‐modifying enzymes (AMEs) such as aminoglycoside phosphotransferases, aminoglycoside acetyltransferases, or aminoglycoside nucleotidyltransferases can also confer aminoglycoside resistance in *P. aeruginosa*. According to the authors Thacharodi and Lamont, aminoglycoside‐resistant strains developed independently through two distinct mechanisms: the MexXY efflux pump and AMEs [66]. Therefore, further studies are required to evaluate antibiotic adjuvant efficacy across a broader range of susceptible and resistant *P. aeruginosa* strains exhibiting diverse resistance mechanisms.

Virtual screening was conducted to assess the interactions of silybin and diclofenac with the selected target proteins. Molecular docking and GROMACS simulation analyses hypothesized two potential target interactions for silybin and diclofenac: LolA (PDB ID: 7Z6W) and the *β*‐sliding clamp of DNA polymerase III (PDB ID: 4K3S), where MM/PBSA analysis indicated that silybin and diclofenac exhibited more favorable total binding free energies than the corresponding reference standards. However, in addition to these in silico analyses, rigorous experimental validation is required to confirm these target involvements and underlying mechanisms.

The in vivo study was performed to evaluate the efficacy of these combinations in a preclinical model. EPEC infection is prevalent worldwide, including in Bangladesh, where it accounts for approximately 30%–40% of infantile diarrhea cases [67]. For this reason, the current study selected the EPEC mouse model in Swiss Albino mice, which provided insight into the efficacy of these combinations in percentage of body weight changes, restoration of total WBC count, alleviation of colitis and edema, histopathological preservation of cells, restoration of stool scoring, and reduction of blood infection. In every section, combination drugs outperformed gentamicin monotherapy. Gentamicin has a relatively narrow therapeutic window and is eliminated predominantly by the kidneys. Its key advantages are bactericidal activity and a postantibiotic effect against serious gram‐negative infections. However, nephrotoxicity remains one of the key obstacles associated with gentamicin use, for which therapeutic drug monitoring is recommended. The clinically recommended starting dose is 7 mg/kg for the general adult population [68]. With respect to silybin, pharmacokinetic evidence indicates that the oral bioavailability of conventional silybin is poor and highly formulation‐dependent. Silybin undergoes extensive phase‐II conjugation, and most of the resulting conjugates are rapidly excreted into bile [69]. Because only 1%–5% of an administered silybin dose is excreted unchanged in urine, in contrast to gentamicin, which undergoes little hepatic metabolism and is eliminated almost entirely via renal excretion, the two agents are unlikely to compete for a shared elimination pathway. It can therefore be hypothesized that no severe pharmacokinetic interaction exists between gentamicin and silybin; however, this hypothesis requires experimental validation. With respect to diclofenac, single oral doses in healthy adults produce Cmax values ranging from 0.001759 to 7.171 mg/L, with substantial variability depending on age, dose, and formulation, where sustained‐release formulations produce lower but measurable systemic concentrations. Diclofenac is extensively bound to plasma proteins and undergoes substantial hepatic metabolism, where approximately 60%–70% of the administered dose is recovered in urine as conjugated metabolites. Because NSAIDs such as diclofenac are associated with nephrotoxicity [70], coadministration of diclofenac with gentamicin raises legitimate clinical safety concerns. However, a study in rats reported that gentamicin (100 mg/kg/day) nephrotoxicity was potentiated following prolonged (27‐day) cotreatment with diclofenac sodium (2.5 mg/kg/day), but not after short‐term (5‐day) cotreatment [71]. In addition to the pharmacokinetics, there is a biological rationale for using the Gen + Sily combination, as silybin is considered to have a favorable safety profile. In studies of silybin across different patient populations, doses ranging from 140 mg twice or thrice daily to 1400 mg per day were coadministered with the main drug; patients experienced no severe toxicity or adverse effects, though mild headache, abdominal pain, and nausea were reported [72]. In addition, silybin has been reported to mitigate gentamicin‐induced nephrotoxicity. Nephrotoxicity induced by a 10‐day administration of gentamicin (200 mg/kg) in mice is associated with oxidative stress, lipid peroxidation, and decreased renal superoxide dismutase activity; the natural antioxidant silybin alleviates these effects [73]. Another study suggested that silybin has a superior protective effect against gentamicin‐induced ototoxicity in guinea pigs [74]. The current study demonstrated the protective effects of silybin, as evidenced by improvements in colitis, edema, and histopathological outcomes, supporting its potential clinical feasibility as an antibiotic adjuvant. However, future studies should include direct assessments of renal biomarkers, cytokine profiles, and oxidative stress markers to comprehensively evaluate the efficacy, safety, and potential toxicity of these combinations.

## 5. Conclusion

In this scientific investigation, the repurposed drugs silybin and diclofenac were evaluated as potential antibiotic adjuvants to address the growing problem of AMR. The current study suggested that these nonantibiotics, when strategically combined with antibiotics, significantly enhanced antibacterial activity, indicating a synergistic therapeutic approach. In silico analysis hypothesized two target proteins that should be rigorously validated through experimental studies. Additionally, further studies on the safety, toxicity, and efficacy of these combinations must be conducted through preclinical and clinical trials.

## Author Contributions

Rafat Hossain Rafi: conceptualization, investigation, methodology, data curation, writing—original draft, and writing—review and editing, formal analysis, funding acquisition, and visualization. Anamul Haque: investigation and formal analysis. Md. Jonaid Tawfiq: writing—original draft, and formal analysis. Chandan Barai: resources and software. Md. Rafiqul Islam Khan: methodology and resources. Md. Omar Faruqe: formal analysis. Mst Sadia Afroz Shoily: formal analysis. Md. Abdul Alim Al‐Bari: conceptualization, supervision, validation, and writing—review and editing.

## Funding

This study was supported by the National Science and Technology (NST) Fellowship 2022‐2023 of the Ministry of Science and Technology, Government of the People′s Republic of Bangladesh (10.13039/501100008804, merit: 413, reg. No: 825).

## Conflicts of Interest

The authors declare no conflicts of interest.

## Supporting information


**Supporting Information** Additional supporting information can be found online in the Supporting Information section. Table S1: Represents FICI values for combinations against susceptible laboratory reference strains. Table S2: Represents FICI values for combinations against gentamicin‐ and amikacin‐resistant bacteria. Table S3: Molecular docking interaction profile of lead drug candidates and standard inhibitors against 4K3S and 7Z6W targets.

## Data Availability

The data that support the findings of this study are available from the corresponding author upon reasonable request.
