# Supplementary material for: Mechanistic Evaluation of LolA and β‐Sliding Clamp Inhibition by Silybin and Diclofenac as Gentamicin Adjuvants Against Enteropathogenic Escherichia coli
Source: Int J Microbiol. 2026 Sep 28;2026:5624539. doi: 10.1155/ijm/5624539 (PMC13620402; doi:10.1155/ijm/5624539)
Supplement: Supplementary file 1 [file IJM-2026-5624539-s001.docx]

**Table S1: Represents FICI values for combinations against susceptible laboratory reference strains.**

| **Combination** | ***P. aeruginosa (ATCC 27853) FICI*** | **Interpretation** | ***E. coli (EPEC) FICI*** | **Interpretation** | ***S. boydii (ATCC 9207) FICI*** | **Interpretation** |
| --- | --- | --- | --- | --- | --- | --- |
| **Oxy+Sily** | 1.0625 | No interaction | 1.0156 | No interaction | 1.0156 | No interaction |
| **Oxy+Dic** | 1.0625 | No interaction | 1.0312 | No interaction | 0.126 | **Synergy** |
| **Doxy+Sily** | 2.0625 | No interaction | 0.3125 | **Synergy** | 0.5078 | No interaction |
| **Doxy+Dic** | 1.0312 | No interaction | 0.0938 | **Synergy** | 0.252 | **Synergy** |
| **Chl+Sily** | 1.125 | No interaction | 0.75 | No interaction | 1.0312 | No interaction |
| **Chl+Dic** | 0.5625 | No interaction | 0.5 | **Synergy** | 1.0156 | No interaction |
| **Cip+Sily** | 1.001 | No interaction | 0.5156 | No interaction | 2.001 | No interaction |
| **Cip+Dic** | 1.001 | No interaction | 0.2656 | **Synergy** | 1.0002 | No interaction |
| **Pen G+Sily** | 2 | No interaction | 3 | No interaction | 0.625 | No interaction |
| **Pen G+Dic** | 2 | No interaction | 4 | No interaction | 0.5625 | No interaction |
| **Ery+Sily** | 1.125 | No interaction | 1.0312 | No interaction | 0.5312 | No interaction |
| **Ery+Dic** | 0.5625 | No interaction | 2.125 | No interaction | 0.1289 | **Synergy** |
| **Azi+Sily** | 0.625 | No interaction | 3 | No interaction | 2.0625 | No interaction |
| **Azi+Dic** | 1.25 | No interaction | 5 | Antagonism | 1.0156 | No interaction |
| **Gen+Sily** | 1.0039 | No interaction | 0.2578 | **Synergy** | 0.0352 | **Synergy** |
| **Gen+Dic** | 1.0039 | No interaction | 0.0664 | **Synergy** | 0.0332 | **Synergy** |

**Table S2: Represents FICI values for combinations against gentamicin and amikacin resistant bacteria.**

| **Combinations** | ***E. coli* FICI** | **Interpretation** | ***K. pneumoniae* FICI** | **Interpretation** | ***P. aeruginosa* FICI** | **Interpretation** |
| --- | --- | --- | --- | --- | --- | --- |
| **Gen+Sily** | 0.3125 | **Synergy** | 0.375 | **Synergy** | 2.5 | No interaction |
| **Gen+Dic** | 0.1875 | **Synergy** | 0.375 | **Synergy** | 2.5 | No interaction |
| **Ami+Sily** | 1.5 | No interaction | 1.5 | No interaction | 2.5 | No interaction |
| **Ami+Dic** | 1 | No interaction | 1.5 | No interaction | 2.5 | No interaction |

**Table S3:** **Molecular docking interaction profile of lead drug candidates and standard inhibitors against 4K3S and 7Z6W targets**

| **Complex** | **Binding Affinity (kcal/mol)** | **Interacting Residue** | **Interaction Type** | **Distance (Å)** |
| --- | --- | --- | --- | --- |
| **4K3S + Standard (CID73010404)** | -5.8 | PRO28 | H-bond | 1.93 |
|  |  | PRO71 | π–σ | 3.93 |
|  |  | PRO71 | Alkyl | 4.2 |
|  |  | ARG73 | Alkyl | 4.45 |
|  |  | PRO25, PRO71 | π–Alkyl | 5.36, 4.94 |
| **4K3S + Diclofenac (Drug)** | -6.4 | ASN295 | Halogen (Cl) | 3.26 |
|  |  | ARG282 | π–cation | 4.31 |
|  |  | ARG282 | π–Alkyl | 5 |
| **7Z6W + Standard (MAC13243)** | -8.7 | PHE47 | π–cation | 4.45 |
|  |  | ILE93 | π–σ | 3.46 |
|  |  | PHE140 | π–π stacked | 5.47 |
|  |  | TYR152 | π–π stacked | 4.31 |
|  |  | VAL13, VAL41 | Alkyl | 4.19, 4.68 |
|  |  | LEU39, LEU154 | Alkyl | 4.30, 4.65 |
|  |  | VAL41, ARG43 | π–Alkyl | 4.07, 5.20 |
|  |  | LEU39, PHE16 | π–Alkyl | 5.39, 4.85 |
|  |  | PHE20, TRP49 | π–Alkyl | 4.39, 5.19 |
|  |  | TYR152 | π–Alkyl | 4.34 |
| **7Z6W + Lipoprotein** | -7.2 | TYR152 | H-bond | 2.44 |
|  |  | GLU144 | H-bond | 2.34 |
|  |  | SER150 | C–H···X | 3.4 |
|  |  | LEU39, VAL41 | Alkyl | 4.98, 4.37 |
|  |  | ILE93, ALA142 | Alkyl | 5.00, 5.01 |
|  |  | LEU154, LEU6 | Alkyl | 4.48, 4.30 |
|  |  | LEU10, LEU115 | Alkyl | 5.19, 4.29 |
|  |  | ILE129, VAL13 | Alkyl | 4.25, 4.26 |
|  |  | PHE20, PHE47 | π–Alkyl | 4.38, 3.92 |
|  |  | TRP49, PHE90 | π–Alkyl | 5.17, 5.35 |
|  |  | PHE127, PHE140 | π–Alkyl | 5.46, 4.33 |
|  |  | TYR152 | π–Alkyl | 5.27 |
| **7Z6W + Silybin (Drug)** | -10.4 | SER14 | H-bond | 2.06 |
|  |  | LYS42 | H-bond | 2.83 |
|  |  | ARG43 | H-bond | 2.68 |
|  |  | TYR152 | H-bond | 2.93 |
|  |  | ILE93 | π–σ | 3.88 |
|  |  | PHE90 | π–π T-shaped | 5.25 |
|  |  | LEU59 | Alkyl | 4.89 |
|  |  | ILE93, VAL41 | π–Alkyl | 5.39, 4.95 |
|  |  | ARG43, PHE47 | π–Alkyl | 4.96, 4.66 |
|  |  | TRP49 | π–Alkyl | 4.15, 5.27 |
